# Impact of perioperative hemodynamic optimization therapies in surgical patients: economic study and meta-analysis

**DOI:** 10.1186/s12871-020-00987-y

**Published:** 2020-03-31

**Authors:** João M. Silva-Jr, Pedro Ferro L. Menezes, Suzana M. Lobo, Flávia Helena S. de Carvalho, Mariana Augusta N. de Oliveira, Francisco Nilson F. Cardoso Filho, Bruna N. Fernando, Maria Jose C. Carmona, Vanessa D. Teich, Luiz Marcelo S. Malbouisson

**Affiliations:** 1 Anesthesiology Department, Barretos Cancer Hospital, PIOXII Foundation, São Paulo, Brazil; 2 Anesthesiology Department, Hospital do Servidor Público Estadual, IAMSPE, São Paulo, Brazil; 3 Anesthesiology Division, Hospital das Clínicas da Faculdade de Medicina da Universidade de São Paulo, São Paulo, Brazil; 4 Hospital Israelita Albert Einstein, São Paulo, Brazil; 5 Hospital de base de São José do Rio Preto, São José do Rio Preto, São Paulo, Brazil

**Keywords:** Surgery, Hemodynamic optimization, Complications, Economic, Cost-effective, Public health system

## Abstract

**Background:**

Several studies suggest that hemodynamic optimization therapies can reduce complications, the length of hospital stay and costs. However, Brazilian data are scarce. Therefore, the objective of this analysis was to evaluate whether the improvement demonstrated by hemodynamic optimization therapy in surgical patients could result in lower costs from the perspective of the Brazilian public unified health system.

**Methods:**

A meta-analysis was performed comparing surgical patients who underwent hemodynamic optimization therapy (intervention) with patients who underwent standard therapy (control) in terms of complications and hospital costs. The cost-effectiveness analysis evaluated the clinical and financial benefits of hemodynamic optimization protocols for surgical patients. The analysis considered the clinical outcomes of randomized studies published in the last 20 years that involved surgeries and hemodynamic optimization therapy. Indirect costs (equipment depreciation, estate and management activities) were not included in the analysis.

**Results:**

A total of 21 clinical trials with a total of 4872 surgical patients were selected. Comparison of the intervention and control groups showed lower rates of infectious (RR = 0.66; 95% CI = 0.58–0.74), renal (RR = 0.68; 95% CI = 0.54–0.87), and cardiovascular (RR = 0.87; 95% CI = 0.76–0.99) complications and a nonstatistically significant lower rate of respiratory complications (RR = 0.82; 95% CI = 0.67–1.02). There was no difference in mortality (RR = 1.02; 95% CI = 0.80–1.3) between groups. In the analysis of total costs, the intervention group showed a cost reduction of R$396,024.83-BRL ($90,161.38-USD) for every 1000 patients treated compared to the control group. The patients in the intervention group showed greater effectiveness, with 1.0 fewer days in the intensive care unit and hospital. In addition, there were 333 fewer patients with complications, with a consequent reduction of R$1,630,341.47-BRL ($371,173.27-USD) for every 1000 patients treated.

**Conclusions:**

Hemodynamic optimization therapy is cost-effective and would increase the efficiency of and decrease the burden of the Brazilian public health system.

## Background

Millions of major surgeries are performed every year worldwide [1]. The mortality and morbidity rates of high-risk surgeries vary among countries but are considered high, making them a global problem [2–4]. In Brazil, in 2008, the mortality rate of patients admitted to intensive care units (ICUs) after major surgeries was 15%; after 90 days, it reached 20.3%. The main postoperative complication found in Brazil was sepsis (24.7%), and the main cause of death was multiple organ dysfunction [5].

Postoperative complications in high-risk patients undergoing major surgery are associated with low cardiorespiratory reserve and the inability to maintain adequate oxygen delivery (O2) during surgical trauma to meet the increased metabolic demand [6–8]. As a consequence, there is an imbalance in the ratio of oxygen delivery to oxygen consumption, leading to hypoperfusion, multiple organ dysfunction and severe infections, which are important causes of postoperative mortality [4]. Perioperative hemodynamic optimization therapy aims to adjust cardiac function to meet the increased demand during the perioperative period, thus avoiding hypovolemia or hypervolemia and, ultimately, tissue hypoperfusion and postoperative complications. This requires adequate hemodynamic monitoring to guide the early treatment of each patient, allowing earlier identification of the need for fluid optimization, blood transfusion, and vasoactive drugs.

Studies and meta-analyses have shown that perioperative hemodynamic optimization therapy has a significant impact on the outcomes of high-risk patients undergoing major surgery, potentially decreasing morbidity and mortality, the length of ICU stay, and the length of hospital stay [9–11]. However, the risk-benefit ratio of this type of monitoring has been questioned because it is invasive and carries risks [12]. Monitoring techniques include pulmonary artery catheterization, transpulmonary thermodilution, echocardiography, transesophageal Doppler echocardiography, pulse contour analysis, partial carbon dioxide rebreathing and bioimpedance.

Cost-effectiveness is represented by a ratio between monetary cost, usually expressed in a national currency, in the numerator and a measure of health gain in the denominator. The poor adherence to perioperative hemodynamic optimization therapy in clinical practice combined with the need to improve the effectiveness of care for surgical patients in the face of increasing demand points to the urgent need for cost-effectiveness assessments that encourage the use of perioperative hemodynamic optimization therapy in this area, particularly in resource-limited settings.

Health resources are increasingly limited, and a lack of knowledge on a method is a barrier to the implementation of new therapies with proven effectiveness that could not only save lives but also lead to a reduction in resource use. Our hypothesis is that these interventions, due to their significant impact on the length of hospital stay and complications, would be cost-effective for the public health system.

This study aimed to evaluate whether the use of perioperative hemodynamic optimization therapy is cost-effective for patients undergoing major surgery from the perspective of the Brazilian unified health system by evaluating the impact of a reduction in the length of hospital stay, complication rates and mortality on hospital costs.

## Methods

A systematic review of the clinical trials indexed in CENTRAL (PubMed), MEDLINE (OvidSP) and EMBASE (OvidSP) between 2001 and 2018 was performed. The search used the following keywords, which were expected to be present in the title and/or abstract: “randomized studies” and “surgeries”, and/or “perioperative”, and/or “high-risk”, and/or “complications”, and/or “intraoperative” and/or “postoperative”, and/or “cardiac output”, and/or “cardiac index”, and/or “hemodynamic monitoring”, and/or “hemodynamic optimization therapy”, and/or “hemodynamic intervention”, and/or “cost-effectiveness”, and/or “mortality”. Methods identical to those recommended by the Cochrane systematic review of randomized controlled trials on increased blood flow to the organs, with explicitly defined goals and results after surgery, were used.

Only studies published in the English language were included. Two independent researchers identified the titles and abstracts of the potentially eligible studies. Disagreements between the investigators were resolved by a consensus. Two other researchers extracted the following data from the full texts of potentially eligible studies: study design, patient population, interventions and outcomes. Similar to the approach used for the selection of texts, any disagreements between researchers regarding the data extraction were resolved by a consensus. Each included study was assessed independently by the first and second reviewers for risk of bias in random sequencing generation, allocation concealment, blinding of participants and personnel, blinding of outcomes assessment, incomplete outcome data, selective reporting, and other sources of bias using the Cochrane Risk of Bias Tool [13]. In the absence of appropriate published data, at least one attempt was made to contact the authors of eligible studies to obtain necessary data. The analysis was performed with the best available information when there was no response.

The following information and outcomes were recorded: number of patients with respiratory complications (i.e., the need for respiratory support for more than 24 h after surgery, hypoxemia, and acute changes in lung mechanics), cardiovascular complications (need for hemodynamic support, such as the use of inotropes and vasopressors during the postoperative period), renal complications (oliguria, unexpected increase in creatinine and need for dialysis) and infectious complications (infections that occurred during the postoperative period) based on the records of each study. Mortality was assessed throughout the longer follow-up period (primary outcome) or was examined as in-hospital mortality. Many studies reported complications as the number of complications rather than the number of patients with complications, but we examined only the last unit in the analysis, which was the number of complications per patient.

To calculate the costs of these complications, only the length of ICU stay was considered.

The inclusion criteria of the selected studies were as follows:

1. studies in adult patients (18 years or older);
2. studies with patients undergoing hemodynamic optimization therapy with some type of cardiac output monitor;
3. studies that related the costs of hemodynamic optimization therapy and its outcomes, such as the reduction in mortality or morbidity rates or the reduction in the length of hospital or ICU stay; and
4. interventional studies comparing the use of invasive or minimally invasive monitoring with the standard strategy for hemodynamic optimization to alter clinical outcomes. The intervention should meet the following criteria:

Perioperative period: The administration of fluids with or without inotropes/vasoactive drugs to increase blood flow (standard therapy group) was compared with goals measured explicitly with invasive or minimally invasive hemodynamic monitoring (intervention group). The perioperative period started at the beginning of surgery and lasted up to 24 h after surgery. Explicit goals were defined for the cardiac index, oxygen delivery (DO_2_), oxygen consumption, systolic volume, mixed or central venous oxygen saturation, oxygen extraction, or serum lactate.

The exclusion criteria were as follows:

1. animal studies;
2. studies published prior to 2001;
3. observational studies that did not use clinical intervention to change outcomes or case reports; and
4. studies involving critically ill patients prior to intervention or with established sepsis who therefore had a high probability of unfavorable outcomes and death regardless of the intervention.

### Cost assessment

For the cost-effectiveness analysis, the costs were separated into 10 categories and two periods: the intraoperative period (monitoring and costs of fluid infusion, inotropes or vasopressors and blood transfusions) and the postoperative period in the ICU, which was maintained at fixed daily rates regardless of the disease, clinical examinations and procedures and depended on postoperative complications, laboratory diagnosis, and the use of antimicrobial and other agents (cardiac support, renal support, physical therapy and imaging). To avoid confounding factors, the costs of the surgical procedure (considering that the surgeries would have the same magnitude), postoperative analgesia and preoperative state were excluded from the final analysis, as were costs related to the hospital infrastructure (electricity, safety system, etc.) and costs related to equipment depreciation, estate and management activities. This approach makes different institutions comparable not in terms of values but in terms of resource utilization. We believe that this simplified economic analysis can provide reliable and interchangeable data.

The overhead costs were estimated from a social perspective (i.e., regardless of who will bear the cost). However, the unit costs of health resources and services were obtained from the national databases of the Brazilian public health system, and therefore, the direct costs represent the costs borne by the payer [14–19].

The incremental cost-effectiveness analysis was based on the difference in costs divided by the difference in survival days in each group.

The results are described in two ways: in Brazilian currency (R$-BRL) and the equivalent in dollars ($-USD) for the present day.

### Statistical analysis

The analyses were performed in Review Manager (RevMan 5.2.8) using fixed effects models with random effects models for comparison. In the fixed effects regression models, the effect of interest is the same in all the studies, and the differences observed are due only to sampling errors. Otherwise, the random effects model assumes that the effect of interest is not the same in all studies, that is, the model incorporates a measure of the variability of effects between different studies. In the latter model, the larger the sample size is, the greater the weight of the study in estimating the meta-analytic measure.

We applied the intention-to-treat method for all analyses. Treatment effects are reported as the relative risk (RR) and confidence interval (95% CI) for clinical variables or as differences in the weighted average (SD) or median for the length of ICU and hospital stays. Empty cells, the result of studies in which no event was observed in one or both arms, were corrected by adding a fixed value (0.5) to all cells with an initial value of zero. The chi-square test was used to assess whether the differences observed in the results were due to chance. A large chi-square (I^2^ statistic) provided evidence of heterogeneity of the intervention effects (indicating that the estimated effect was beyond chance).

## Results

**Fig. 1:**
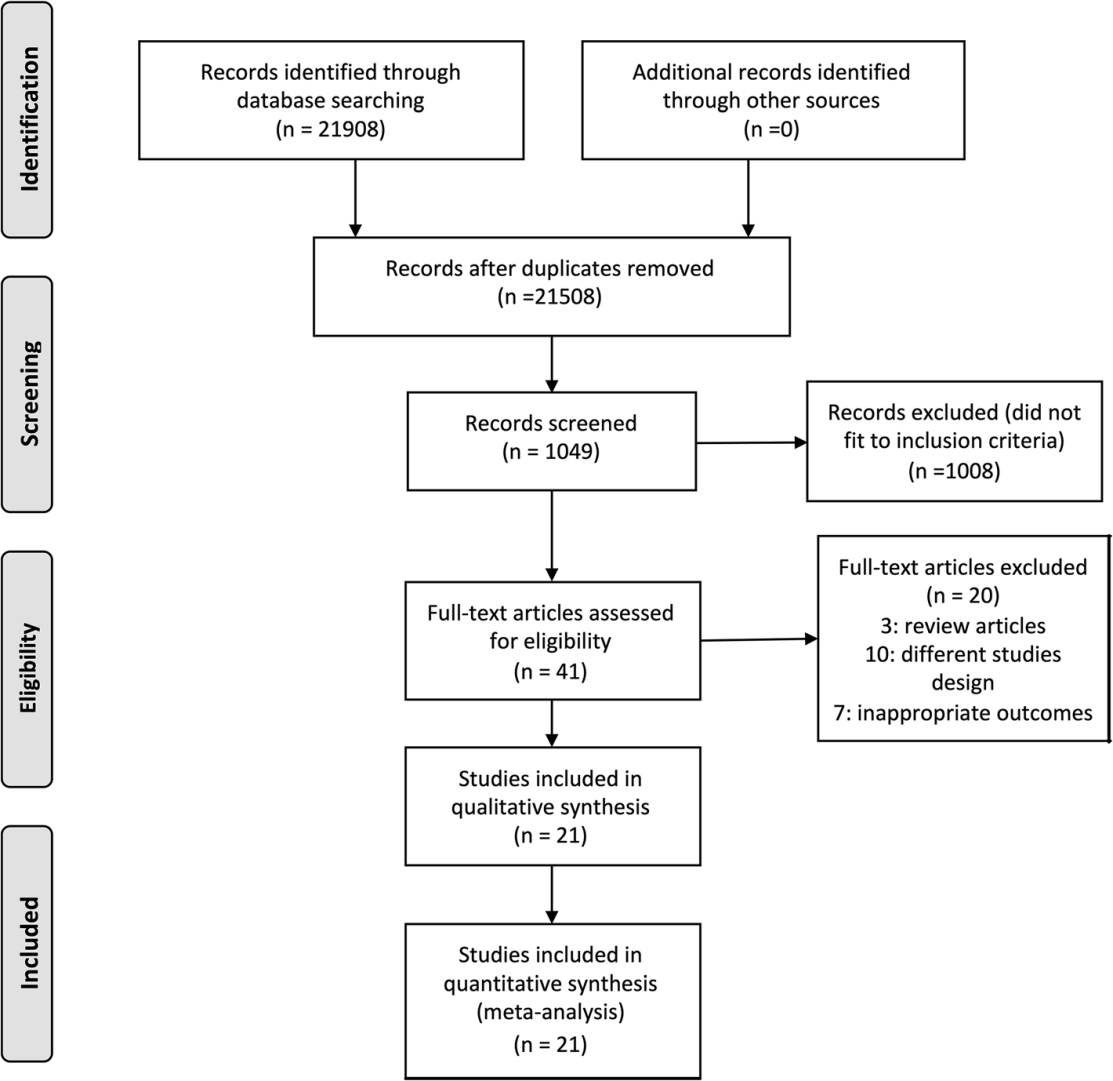
Flow Diagram. PRISMA diagram showing the inclusion and exclusion processes used for the literature search and review

Initially, 52 potential articles were identified. After the inclusion and exclusion criteria were applied, 21 articles remained (Fig. 1).

Of the selected studies, 21 used techniques to measure cardiac output; these studies had a total of 4872 patients at high surgical risk (Table 1).

**Table 1:** Selected studies and the technology used for hemodynamic monitoring

Author-year (ref)	Objective	Technology
Bonazzi et al., 2002 [20]	Evaluation of the impact of hemodynamic optimization using a pulmonary artery catheter on the outcome of patients undergoing vascular surgery.	Pulmonary artery catheter
Venn et al., 2002 [21]	Evaluation of hemodynamic optimization therapy in patients undergoing hip surgery.	Transesophageal Doppler
Conway et al., 2002 [22]	Randomized study to evaluate the influence of fluid titration using transesophageal Doppler during intestinal surgeries.	Transesophageal Doppler
Gan et al., 2002 [23]	Evaluation of the impact of hemodynamic optimization therapy on the reduced hospital stay after major surgeries.	Transesophageal Doppler
Sandham et al., 2003 [24]	Randomized study evaluating the use of pulmonary artery catheters in high-risk surgical patients.	Pulmonary artery catheter
Wakeling et al., 2005 [25]	Evaluation of transesophageal echocardiography-guided hemodynamic optimization therapy for the reduced hospital stay during the postoperative period of major abdominal surgeries.	Transesophageal Doppler
Pearse et al., 2005 [26]	Evaluation of the use of GDT in highly complex surgeries to reduce perioperative complications and the length of hospital stay.	LiDCO monitoring system
Lobo et al., 2006 [27]	Investigation of the effects of the optimization of oxygen delivery in elective surgeries for high-risk patients.	Pulmonary artery catheter
Noblett et al., 2006 [28]	Evaluation of transesophageal echocardiography-guided hemodynamic optimization therapy in terms of the outcomes of patients undergoing colectomy.	Transesophageal Doppler
Harten et al., 2008 [29]	Randomized study evaluating the effect of hemodynamic optimization on renal function in patients undergoing emergency laparotomy.	FloTrac Vigileo system
Kapoor et al., 2008 [30]	Evaluation of GDT in patients undergoing moderate- to high-risk cardiac surgery.	FloTrac Vigileo system
Mayer et al., 2010 [31]	Evaluation of GDT based on the monitoring of the blood pressure wave in high-risk surgical patients.	FloTrac Vigileo system
Benes et al., 2010 [32]	Evaluation of hemodynamic optimization by fluid loading based on data obtained by Vigileo.	FloTrac Vigileo system
Cecconi et al., 2011 [11]	Evaluation of hemodynamic optimization therapy for patients undergoing total hip arthroplasty under regional anesthesia.	FloTrac Vigileo system
Lobo et al., 2011 [33]	Evaluation of restrictive or conventional strategies for crystalloid administration during GDT in high-risk surgical patients.	LiDCO monitoring system
Salzwedel et al., 2013 [34]	Randomized study evaluating GDT based on the variation in the radial arterial pulse and the cardiac index and the effects of GDT on the postoperative complications of major abdominal surgeries.	FloTrac Vigileo system
van Beest et al., 2014 [35]	Evaluation of the effect of the tissue oxygenation optimization-based protocol on perioperative complication rates.	FloTrac Vigileo system
Pearse et al., 2014 [36]	Evaluation of the clinical effectiveness of the perioperative use of the cardiac output-guided hemodynamic therapy algorithm.	LiDCO monitoring system
Cannesson et al., 2015 [37]	Evaluation of the effects of the systematic implementation of GDT on the length of hospital stay and the incidence of complications after high-risk abdominal surgeries.	EV 1000 (Edwards Lifesciences, Irvine, CA, USA)
Kumar et al., 2015 [38]	Randomized study evaluating the impact of GDT on the cardiac index and O2 extraction rate in patients undergoing abdominal surgery.	FloTrac Vigileo system
Calvo-Vecino et al., 2018 [39]	Randomized study evaluating the impact of GDT on the outcome in patients undergoing major surgeries compared to controls.	Transesophageal Doppler

*GDT* Goal-directed therapy, *O2* Oxygen

**Fig. 2:**
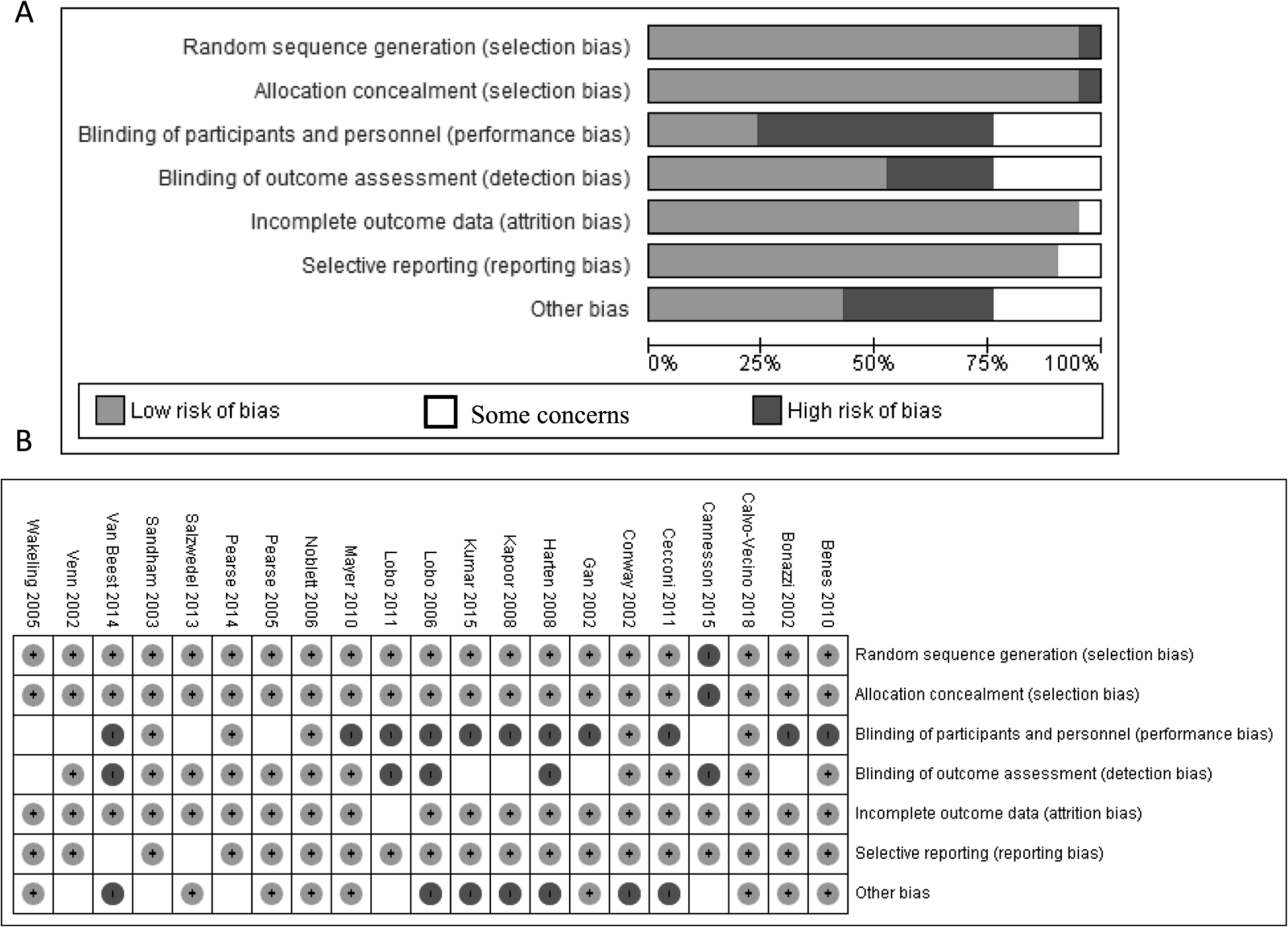
Summary of Risk of Bias Assessment. **a** Risk of bias summary for each included study. **b** Summary of domains for risk of bias assessment of the included studies

The risk of bias assessment for each of the included studies can be visualized (Fig. 2a and b). Most studies reported problems with blinding.

**Fig. 3:**
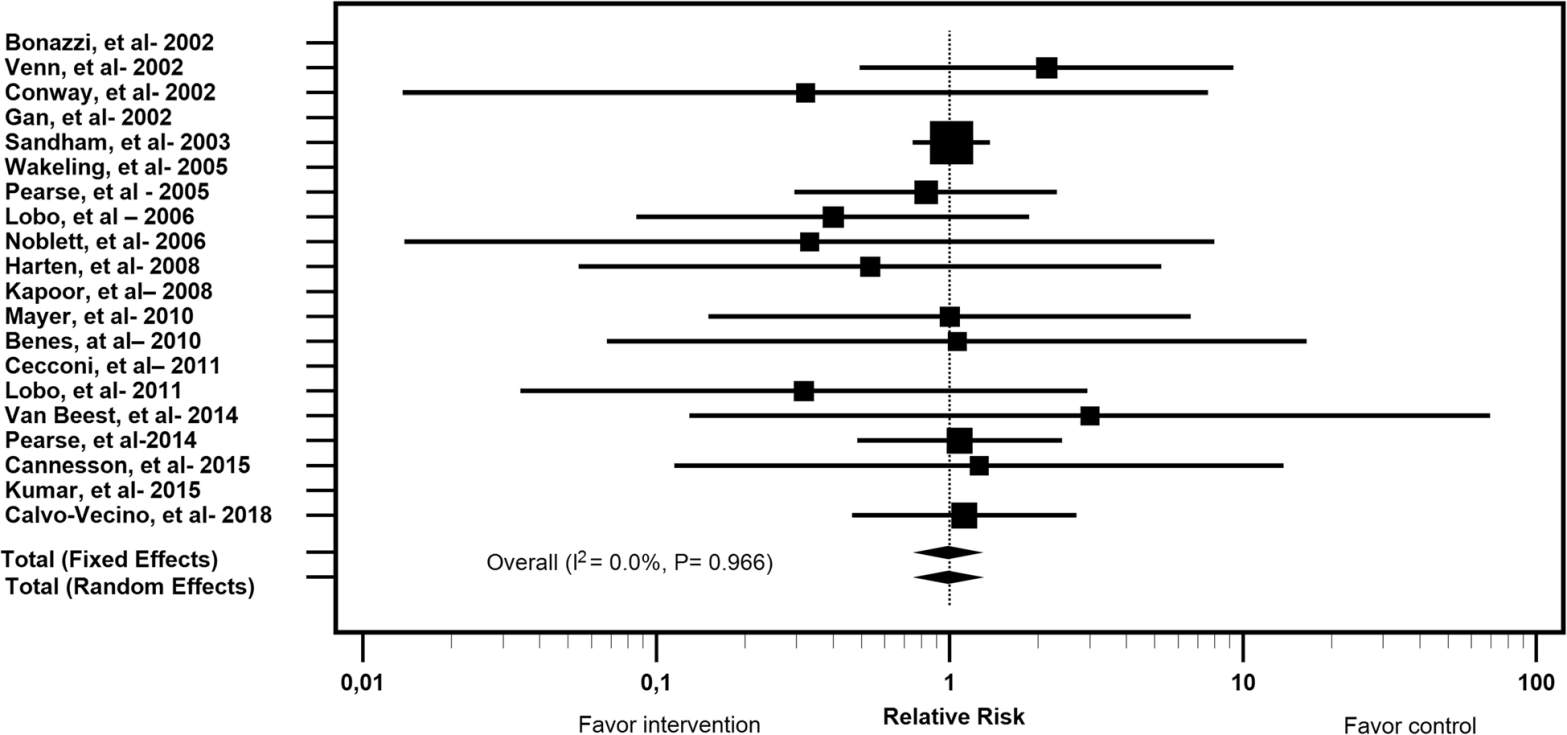
Forest Plot for Mortality in the Intervention and Control Groups

The mortality rate in the intervention group was 2.6% (125); in the control group, it was 2.5% (122). There were no statistically significant differences in mortality rates (Fig. 3).

**Fig. 4:**
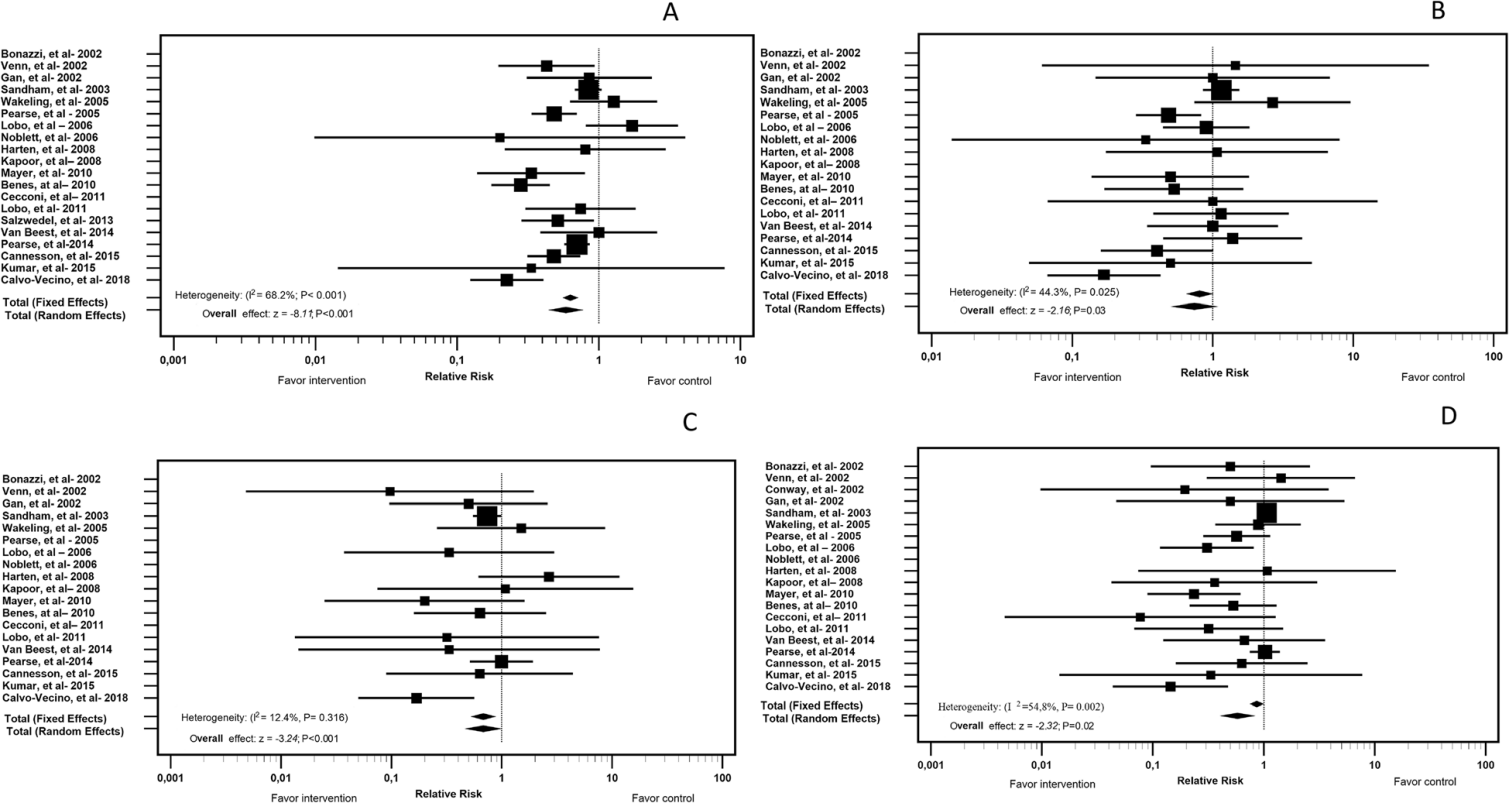
Forest Plot of Complications in the ICU; Comparison Between the Intervention and Control Groups. **a** - infectious; **b** - respiratory; **c** - renal; **d** - cardiovascular

The rates of infectious complications (RR = 0.66, 95% CI = 0.58–0.74), renal complications (RR = 0.68, 95% CI = 0.54–0.87) and cardiovascular complications (RR = 0.87, 95% CI = 0.76–0.99) after surgery were significantly higher in the control group than in the intervention group. Regarding respiratory complications, there was no significant difference in the random effects analysis, but the fixed effects analysis yielded statistically significant differences (RR = 0.81; 95% CI = 0.66–0.98) (Fig. 4).

In the comparison between groups, the patients in the intervention group had shorter lengths of hospital and ICU stays (Table 2).

**Table 2:** Comparison of the variables analyzed in determining the costs of the intervention and standard therapy groups

Variables	Number of studies involved (total number of patients)	Intervention group	Standard therapy group	RR (95% CI)
**Intraoperative period**				
Use of vasoactive drugs	15 (*N* = 4104)	*N* = 785 (19.1%)	*N* = 609 (14.8%)	1.24 (1.13–1.37)
Crystalloid fluids (mL); median (min-max)	17 (*N* = 2681)	3000 (1000–6713)	2558 (1286–6200)	
Colloid fluids (mL); median (min-max)	18 (*N* = 4393)	1188 (0–2426)	817 (0–2236)	
Blood products (mL); median (min-max)	15 (*N* = 4420)	244.5 (0–825)	267 (0–975)	
**Complications (postoperative period)**	21 (*N* = 4872)	1017 (20.9%)	1350 (27.7%)	0.75 (0.70–0.81)
Infectious complications	17 (*N* = 4530)	*N* = 384 (8.4%)	*N* = 581 (12.8%)	0.66 (0.58–0.74)
Respiratory complications	19 (*N* = 4655)	*N* = 160 (3.4%)	*N* = 193 (4.1%)	0.82 (0.67–1.02)
Renal complications	19 (*N* = 4655)	*N* = 108 (2.3%)	*N* = 158 (3.4%)	0.68 (0.54–0.87)
Cardiovascular complications	21 (*N* = 4872)	*N* = 365 (7.4%)	*N* = 418 (8.5%)	0.87 (0.76–0.99)
**Length of ICU stay (days)**; **median (min-max)**	14 (*N* = 1637)	1.9 (0–5)	2.9 (0–5)	
**Mortality rate**	20 (*N* = 4712)	*N* = 125 (2.6%)	*N* = 122 (2.5%)	1.02 (0.80–1.31)
**Length of hospital stay (days)**; **median (min-max)**	20 (*N* = 4852)	10 (5–20)	11 (6–20)	

*RR* Relative risk compared to the intervention group, *CI* Confidence interval, *ICU* Intensive care unit, *N* Total number of patients, *U* Units

Costs were calculated based on the costs for the intraoperative (anesthesia, monitoring, infusions and blood products) and postoperative (patient care, clinical exams, routine procedures, routine laboratory and radiological exams and others) periods. Each outcome was accounted for with costs associated with that particular complication. The mean outcomes were calculated by multiplying the output by the probability of being in that particular health state and the length of days in the ICU. The total cost assumes that every patient (intervention or control group) had all complications (Tables 3 and 4).

**Table 3:** Pattern of resource use by each patient treated with an intervention

Resource	Quantity	Unit cost	Weighted cost
Intraoperative period			
Monitoring of cardiac output (average of prices considering only one-time-use disposable devices^a^)	1	R$1363.00	R$1363.00
Infusion of medications (risk of inotropes and vasopressors per patient)	1.24	R$ 34.50	R$42.78
Infusion of crystalloid fluids (per L)	3.0 L	R$10.36	R$31.08
Infusion of colloid fluids (per L)	1.188 L	R$90.00	R$106.92
Transfusions of blood products (per unit)	1.22 IU	R$553.30	R$675.02
**Subtotal - intraoperative period**	**–**	**–**	**R$2218.80-BRL ($505.14-USD)**
Hospital stay			
Hospital daily rate	10 days	R$310.00	R$3100.00
ICU daily rate	1.9 days	R$1138.00	R$2162.20
**Subtotal - hospital stay**	**–**	**–**	**R$5262.20-BRL ($1198.02-USD)**
Treatment of complications (based on the length of ICU stay)			
Infectious complications (treatment of sepsis)	1.9 days	R$1868.00	R$3549.2
Respiratory complications (including mechanical ventilation)	1.9 days	R$1202.35	R$2284.46
Renal complications (including days on dialysis)	1.9 days	R$1449.42	R$2753.89
Cardiovascular complications (including visits during the 1st postoperative period and the infusion of vasopressors)	1.9 days	R$1252.70	R$2380.13
**Subtotal - treatment of complications**	**–**	**–**	**R$10,967.68-BRL ($2496.97-USD)**
**Total - intervention**	**–**	**–**	**R$18,448.68-BRL ($4200.14-USD)**

^a^One-time-use or disposable devices: catheters and probes acquired by the hospital in certain quantities; the debt is borrowed by the hospital

**Table 4:** Pattern of resource use by each patient treated with standard therapy

Resource	Quantity	Unit cost	Weighted cost
Intraoperative period			
Monitoring of cardiac output	0	R$1363.00	0
Infusions of medications (risk of inotropes and vasopressors per patient)	1	R$34.50	R$34.50
Infusion of crystalloid fluids (per L)	2.558 L	R$10.36	R$26.50
Infusion of colloid fluids (per L)	0.817 L	R$90.00	R$73.53
Transfusions of blood products (per unit)	1.33 IU	R$553.30	R$735.88
**Subtotal - intraoperative period**	**–**	**–**	**R$870.41-BRL ($198.16-USD)**
Hospital stay			
Hospital daily rate	11 days	R$310.00	R$3410.00
ICU daily rate	2.9 days	R$1138.00	R$3300.2
**Subtotal - hospital stay**	**–**	**–**	**R$6710.20-BRL ($1527.68-USD)**
Treatment of complications (based on the length of ICU stay)			
Infectious complications (treatment of sepsis)	2.9 days	R$1868.00	R$5417.2
Respiratory complications (including mechanical ventilation)	2.9 days	R$1202.35	R$3486.81
Renal complications (including dialysis)	2.9 days	R$1449.42	R$4203.31
Cardiovascular complications (including visits during the 1st postoperative period and the infusion of vasopressors)	2.9 days	R$1252.70	R$3632.83
**Subtotal - treatment of complications**	**–**	**–**	**R$16,740.15-BRL ($3811.16-USD)**
**Total - standard therapy**	**–**	**–**	**R$24,320.76-BRL ($5537.00-USD)**

The amount was extracted from the findings of the studies (Table 2) and extrapolated to represent the costs of 1 patient from a Brazilian cost perspective.

The intervention group was monitored hemodynamically during the intraoperative period and was managed according to the data measured. The control group did not undergo hemodynamic monitoring and was treated according to the standard procedure.

Regarding complications in terms of only the length of ICU stay, those who received standard therapy showed higher costs when they developed infectious, renal, cardiovascular and respiratory complications (Fig. 4).

**Fig. 5:**
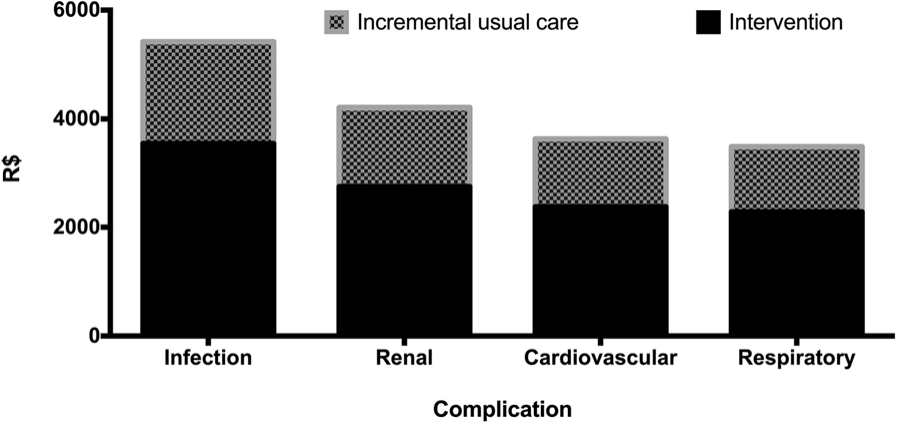
Incremental Cost-Effectiveness Ratio (ICER) per Patient Based on Complications. (Brazilian Reais. R$)

Based on these calculations, when estimating costs for the two types of treatment per 1000 patients, the highest costs were observed for the standard treatment in the presence of complications. On the other hand, the benefits were not sustained for patients without complications or those who did not survive because the costs were higher for patients who were treated with goal-directed therapy (Fig. 5).

**Fig. 6:**
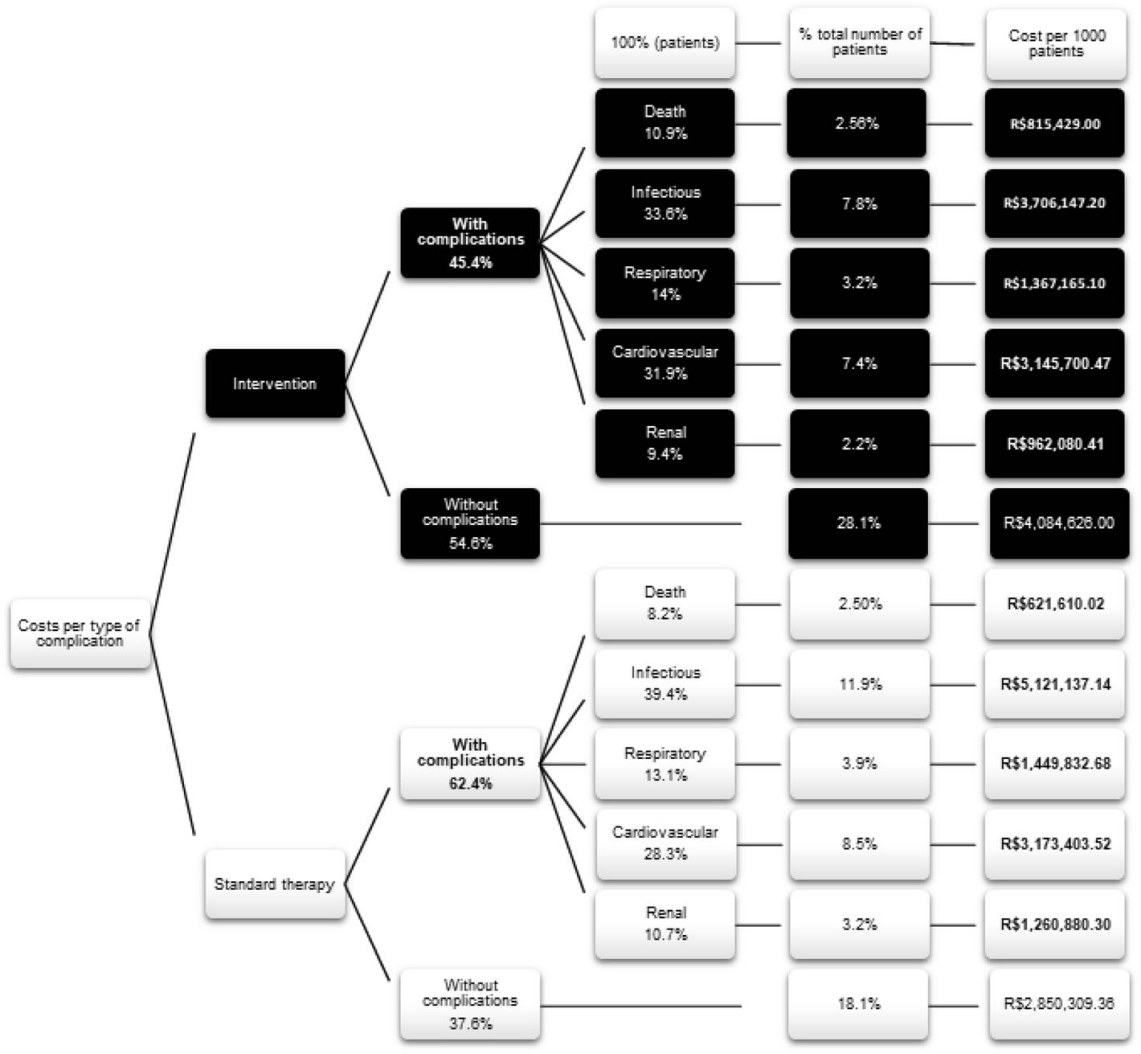
Comparison of ICU Stay-Related Costs per 1000 Patients in the Intervention and Control Groups. (Brazilian Reais. R$)

**Fig. 7:**
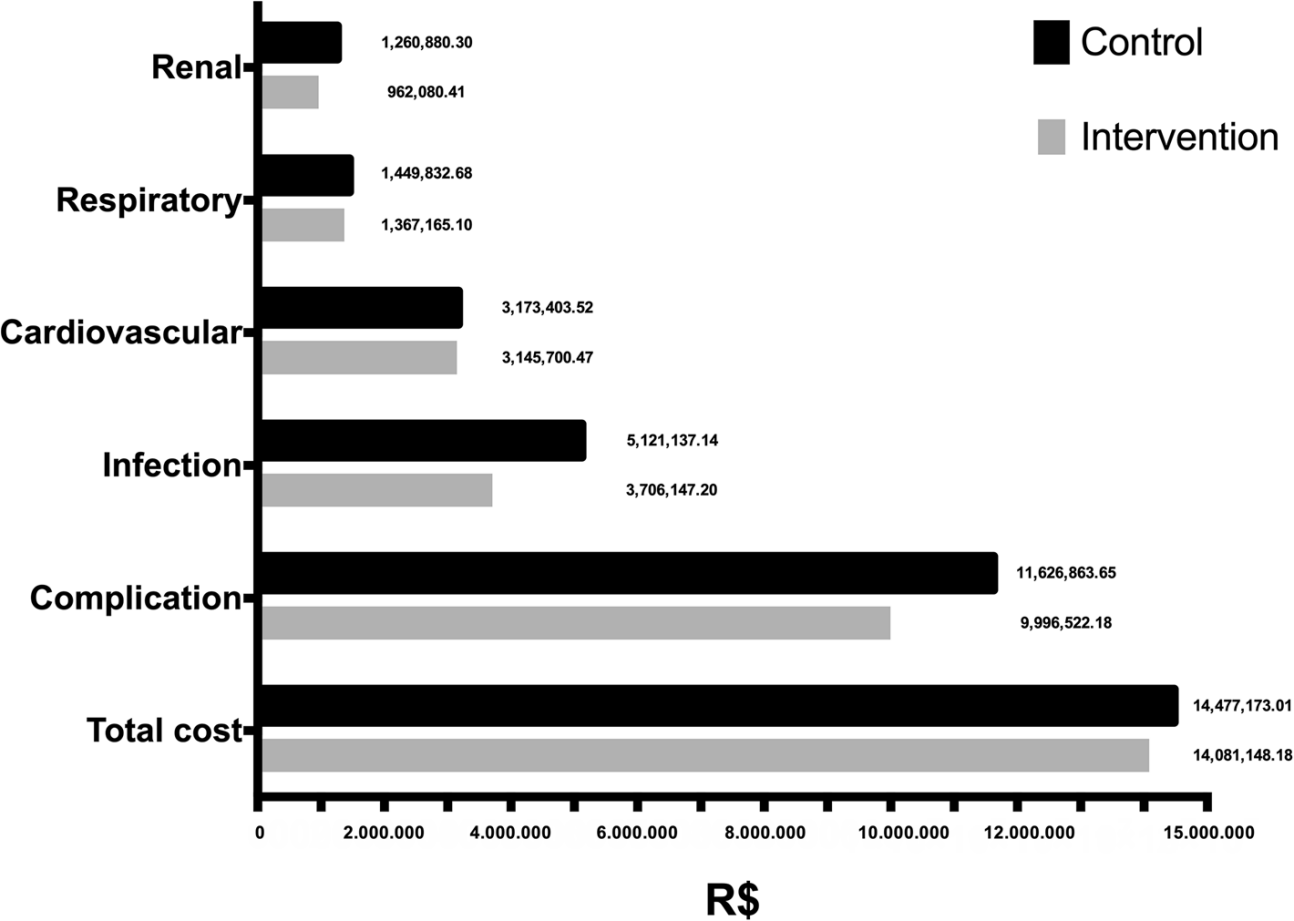
Comparison of Complication-Associated Costs per 1000 Patients in the Intervention and Control Groups. (Brazilian Reais. R$)

The cost and final effectiveness of the intervention group (optimization therapy) were lower than those of the control group (conventional therapy). Although there was no significant difference in mortality rates, the cost of patients in the intervention group based on 1000 treated patients was R$396,024.83-BRL ($90,161.38-USD) less than that of patients in the standard therapy group (incremental). In addition, the intervention group showed a reduction of R$1,630,341.47-BRL ($371,173,27-USD) per 1000 patients with complications (Figs. 6 and 7). A wide difference in costs can be seen for patients with infection (Fig. 7).

## Discussion

This study presented the first cost analysis of perioperative hemodynamic optimization therapy in Brazil. Despite the increase in costs due to the acquisition of materials and equipment and the provision of greater care in the perioperative period, perioperative hemodynamic optimization therapy showed more benefits and lower final costs than standard therapy for high-risk surgical patients.

The benefits of perioperative hemodynamic monitoring, especially in terms of complications and the length of hospital stay, are known [40, 41], but few studies have shown benefits in terms of mortality rates [42]. The data used to perform the calculations did not show differences in mortality rates, but when we performed a cost-effectiveness analysis according to complications, we observed a significant reduction in costs (total cost of standard therapy, $5537.00-USD, versus $4200.14-USD for the intervention) and gains in effectiveness (1.0 days in the ICU and hospital) in terms of the reduced length of stay. These facts translate to an incremental cost-effectiveness ratio of $1336.87-USD to the health system for each patient who receives only standard therapy if presents with complications.

The results of this analysis are consistent with the economic health analyses performed in previous studies. A cost-effectiveness analysis using data from two recent clinical trials of perioperative hemodynamic therapy suggested that this treatment resulted in a net reduction in health costs and therefore was cost-effective [43, 44]. However, more recent data from clinical trials have suggested that this treatment could offer only modest improvements in patient outcomes and, in some cases, provided evidence of borderline clinical effectiveness [36].

The first economic study conducted during the perioperative period was performed by Guest et al. [44], and demonstrated that high-risk patients undergoing a goal-directed hemodynamic optimization strategy protocol experienced not only survival benefits but also lower total hospital costs (median of $10,968.50-USD vs $13,084.90-USD). This analysis did not include the clinical benefit and therefore could be considered a pure cost minimization study. Likewise, subsequent studies have shown cost savings when goal-directed therapy is initiated in the preoperative period [45] or perioperative period [46]. Fenwick et al. [43] performed a cost-effectiveness analysis of a preoperative optimization regimen. In their analysis, the incremental costs were compared to the survival benefits among the different treatment groups. Previous data from other countries have shown similar estimates in elderly patients undergoing hip fracture repair, which is considered a high-cost surgery [47].

Although the results of economic health simulations continue to suggest that perioperative hemodynamic therapy may be economical and, therefore, cost-effective, these findings are sensitive to the size of the treatment effect [45, 46]. Interestingly, the economic evaluations of early hemodynamic therapy in patients with severe sepsis also indicate that treatment can be cost-effective [48, 49]. However, these analyses were based on the assumption of a strong treatment effect, while the results of recent randomized clinical trials suggested that such protocols have little or no clinical benefit in patients with severe sepsis [50].

Thus, while the findings of the current study are consistent with those of previous analyses, definitive evidence provided by a large clinical effectiveness trial is necessary to confirm the economic impact of perioperative hemodynamic cardiac output optimization therapy. However, we emphasize that the findings are important because they show that perioperative hemodynamic intervention represents an excellent expenditure of public money from the Brazilian perspective.

However, this study has some limitations that we must discuss, such as the fact that the cost-effectiveness analysis was based on data from clinical trials with extrapolated results of studies performed in both developed and developing countries, and the type, incidence of complications and length of stay for the same procedure can differ between countries. Despite the use of appropriate methods and long-term survival data, the cost assessment was carried out in a developing country (Brazil), in which it is highly representative of similar countries with the same economic potential. The cost of uncertainty in decision-making was calculated along with the traditional cost-effectiveness results. Cost data and results were collected only during ICU stays, and the cost-benefit analysis may involve other assumptions that could not be extrapolated with these data. Some follow-up data were missing and were resolved with an approximation by multiple imputation [51]. Furthermore, although economic health data were prospectively included in the dataset, the calculations were based on clinical results. These results should be interpreted considering that most tests are designed to detect differences in clinical effectiveness rather than cost-effectiveness. The costs of health care are more variable than clinical results, and economic evaluations may therefore lack statistical power. The primary objective of an economic evaluation is not to test hypotheses but rather to estimate incremental measures of cost-effectiveness and to provide an adequate representation of the uncertainty around these estimates.

Above all, these findings will inform a new clinical policy regarding priorities for future research. Cost-effectiveness analysis is a useful tool for assisting in decision-making regarding the allocation of resources for the appropriate treatment of surgical patients.

## Conclusions

Perioperative hemodynamic optimization therapy is cost effective and indicated in high-risk surgical patients with high chances of developing postoperative complications, reducing the burden on the Brazilian public health system.

## Data Availability

The datasets used and/or analyzed during the current study are available from the corresponding author on reasonable request.

## Abbreviations

RR: Relative risk
CI: Confidence interval
ICU: Intensive care unit
DO2: Oxygen delivery
SD: Standard deviation
GDT: Goal-directed therapy
O2: Oxygen
N: Total number of patients
U: Units
ICER: Incremental Cost-Effectiveness Ratio

## References

[1] Weiser TG, Regenbogen SE, Thompson KD, Haynes AB, Lipsitz SR, Berry WR, Gawande AA (2008). An estimation of the global volume of surgery: a modelling strategy based on available data. Lancet.

[2] Haynes AB, Weiser TG, Berry WR, Lipsitz SR, Breizat AH, Dellinger EP, Herbosa T, Joseph S, Kibatala PL, Lapitan MC (2009). A surgical safety checklist to reduce morbidity and mortality in a global population. N Engl J Med.

[3] Juul AB, Wetterslev J, Gluud C, Kofoed-Enevoldsen A, Jensen G, Callesen T, Norgaard P, Fruergaard K, Bestle M, Vedelsdal R (2006). Effect of perioperative beta blockade in patients with diabetes undergoing major non-cardiac surgery: randomised placebo controlled, blinded multicentre trial. BMJ.

[4] Khuri SF, Henderson WG, DePalma RG, Mosca C, Healey NA, Kumbhani DJ (2005). Participants in the VANSQIP: determinants of long-term survival after major surgery and the adverse effect of postoperative complications. Ann Surg.

[5] Lobo SM, Rezende E, Knibel MF, Silva NB, Paramo JA, Nacul F, Mendes CL, Assuncao M, Costa Filho RC, Grion CC (2008). Epidemiology and outcomes of non-cardiac surgical patients in Brazilian intensive care units. Rev Bras Ter Intensiva.

[6] Bowdle TA (2002). Complications of invasive monitoring. Anesthesiol Clin North Am.

[7] Bennett-Guerrero E, Hyam JA, Shaefi S, Prytherch DR, Sutton GL, Weaver PC, Mythen MG, Grocott MP, Parides MK (2003). Comparison of P-POSSUM risk-adjusted mortality rates after surgery between patients in the USA and the UK. Br J Surg.

[8] Bender JS, Smith-Meek MA, Jones CE (1997). Routine pulmonary artery catheterization does not reduce morbidity and mortality of elective vascular surgery: results of a prospective, randomized trial. Ann Surg.

[9] Kern JW, Shoemaker WC (2002). Meta-analysis of hemodynamic optimization in high-risk patients. Crit Care Med.

[10] Cecconi M, Corredor C, Arulkumaran N, Abuella G, Ball J, Grounds RM, Hamilton M, Rhodes A (2013). Clinical review: goal-directed therapy-what is the evidence in surgical patients? The effect on different risk groups. Crit Care.

[11] Cecconi M, Fasano N, Langiano N, Divella M, Costa MG, Rhodes A, Della Rocca G (2011). Goal-directed haemodynamic therapy during elective total hip arthroplasty under regional anaesthesia. Crit Care.

[12] Benes J, Zatloukal J, Simanova A, Chytra I, Kasal E (2014). Cost analysis of the stroke volume variation guided perioperative hemodynamic optimization - an economic evaluation of the SVVOPT trial results. BMC Anesthesiol.

[13] Higgins JP, Altman DG, Gotzsche PC, Juni P, Moher D, Oxman AD, Savovic J, Schulz KF, Weeks L, Sterne JA (2011). The Cochrane Collaboration's tool for assessing risk of bias in randomised trials. BMJ.

[14] DATASUS. Informatics Department of the Brazilian Public Healthcare System. http://tabnet.datasus.gov.br/cgi/deftohtm.exe?sih/cnv/qiuf.def.

[15] SIGTAP.Management System of the Table of Procedures, Medications and Devices of SUS. http://sigtap.datasus.gov.br/tabela-unificada/app/sec/inicio.jsp.

[16] Ministério da Saúde. Brazilian Healthcare Database: Banco de Preços em Saúde. http://portalsaude.saude.gov.br/index.php/cidadao/principal/banco-de-precos-em-saude?layout=edit&id=86672015.

[17] Sogayar AM, Machado FR, Rea-Neto A, Dornas A, Grion CM, Lobo SM, Tura BR, Silva CL, Cal RG, Beer I, et al. A multicentre, prospective study to evaluate costs of septic patients in Brazilian intensive care units. Pharmacoeconomics. 2008;26(5):425–34. 10.2165/00019053-200826050-00006 18429658

[18] Price Index for Medical Procedures: CBHPM [http://cbr.org.br/wp-content/uploads/2013/05/COMUNICADO-CBHPM-2015-2016.pdf].

[19] Development and Management. Prices Panel [http://paineldeprecos.planejamento.gov.br/analise-materiais].

[20] Bonazzi M, Gentile F, Biasi GM, Migliavacca S, Esposti D, Cipolla M, Marsicano M, Prampolini F, Ornaghi M, Sternjakob S (2002). Impact of perioperative haemodynamic monitoring on cardiac morbidity after major vascular surgery in low risk patients. A randomised pilot trial. Eur J Vasc Endovasc Surg.

[21] Venn R, Steele A, Richardson P, Poloniecki J, Grounds M, Newman P (2002). Randomized controlled trial to investigate influence of the fluid challenge on duration of hospital stay and perioperative morbidity in patients with hip fractures. Br J Anaesth.

[22] Conway DH, Mayall R, Abdul-Latif MS, Gilligan S, Tackaberry C (2002). Randomised controlled trial investigating the influence of intravenous fluid titration using oesophageal Doppler monitoring during bowel surgery. Anaesthesia.

[23] Gan TJ, Soppitt A, Maroof M, el Moalem H, Robertson KM, Moretti E, Dwane P, Glass PS (2002). Goal-directed intraoperative fluid administration reduces length of hospital stay after major surgery. Anesthesiology.

[24] Sandham JD, Hull RD, Brant RF, Knox L, Pineo GF, Doig CJ, Laporta DP, Viner S, Passerini L, Devitt H (2003). A randomized, controlled trial of the use of pulmonary-artery catheters in high-risk surgical patients. N Engl J Med.

[25] Wakeling HG, McFall MR, Jenkins CS, Woods WG, Miles WF, Barclay GR, Fleming SC (2005). Intraoperative oesophageal Doppler guided fluid management shortens postoperative hospital stay after major bowel surgery. Br J Anaesth.

[26] Pearse R, Dawson D, Fawcett J, Rhodes A, Grounds RM, Bennett ED (2005). Early goal-directed therapy after major surgery reduces complications and duration of hospital stay. A randomised, controlled trial [ISRCTN38797445]. Crit Care.

[27] Lobo SM, Lobo FR, Polachini CA, Patini DS, Yamamoto AE, de Oliveira NE, Serrano P, Sanches HS, Spegiorin MA, Queiroz MM (2006). Prospective, randomized trial comparing fluids and dobutamine optimization of oxygen delivery in high-risk surgical patients [ISRCTN42445141]. Crit Care.

[28] Noblett SE, Snowden CP, Shenton BK, Horgan AF (2006). Randomized clinical trial assessing the effect of Doppler-optimized fluid management on outcome after elective colorectal resection. Br J Surg.

[29] Harten J, Crozier JE, McCreath B, Hay A, McMillan DC, McArdle CS, Kinsella J (2008). Effect of intraoperative fluid optimisation on renal function in patients undergoing emergency abdominal surgery: a randomised controlled pilot study (ISRCTN 11799696). Int J Surg.

[30] Kapoor PM, Kakani M, Chowdhury U, Choudhury M, Lakshmy KU (2008). Early goal-directed therapy in moderate to high-risk cardiac surgery patients. Ann Card Anaesth.

[31] Mayer J, Boldt J, Mengistu AM, Rohm KD, Suttner S (2010). Goal-directed intraoperative therapy based on autocalibrated arterial pressure waveform analysis reduces hospital stay in high-risk surgical patients: a randomized, controlled trial. Crit Care.

[32] Benes J, Chytra I, Altmann P, Hluchy M, Kasal E, Svitak R, Pradl R, Stepan M (2010). Intraoperative fluid optimization using stroke volume variation in high risk surgical patients: results of prospective randomized study. Crit Care.

[33] Lobo SM, Ronchi LS, Oliveira NE, Brandao PG, Froes A, Cunrath GS, Nishiyama KG, Netinho JG, Lobo FR (2011). Restrictive strategy of intraoperative fluid maintenance during optimization of oxygen delivery decreases major complications after high-risk surgery. Crit Care.

[34] Salzwedel C, Puig J, Carstens A, Bein B, Molnar Z, Kiss K, Hussain A, Belda J, Kirov MY, Sakka SG (2013). Perioperative goal-directed hemodynamic therapy based on radial arterial pulse pressure variation and continuous cardiac index trending reduces postoperative complications after major abdominal surgery: a multi-center, prospective, randomized study. Crit Care.

[35] van Beest PA, Vos JJ, Poterman M, Kalmar AF, Scheeren TW (2014). Tissue oxygenation as a target for goal-directed therapy in high-risk surgery: a pilot study. BMC Anesthesiol.

[36] Pearse RM, Harrison DA, MacDonald N, Gillies MA, Blunt M, Ackland G, Grocott MP, Ahern A, Griggs K, Scott R (2014). Effect of a perioperative, cardiac output-guided hemodynamic therapy algorithm on outcomes following major gastrointestinal surgery: a randomized clinical trial and systematic review. JAMA.

[37] Cannesson M, Ramsingh D, Rinehart J, Demirjian A, Vu T, Vakharia S, Imagawa D, Yu Z, Greenfield S, Kain Z (2015). Perioperative goal-directed therapy and postoperative outcomes in patients undergoing high-risk abdominal surgery: a historical-prospective, comparative effectiveness study. Crit Care.

[38] Kumar L, Kanneganti YS, Rajan S (2015). Outcomes of implementation of enhanced goal directed therapy in high-risk patients undergoing abdominal surgery. Indian J Anaesth.

[39] Calvo-Vecino JM, Ripolles-Melchor J, Mythen MG, Casans-Frances R, Balik A, Artacho JP, Martinez-Hurtado E, Serrano Romero A, Fernandez Perez C, Asuero de Lis S (2018). Effect of goal-directed haemodynamic therapy on postoperative complications in low-moderate risk surgical patients: a multicentre randomised controlled trial (FEDORA trial). Br J Anaesth.

[40] Hamilton MA, Cecconi M, Rhodes A (2011). A systematic review and meta-analysis on the use of preemptive hemodynamic intervention to improve postoperative outcomes in moderate and high-risk surgical patients. Anesth Analg.

[41] Gurgel ST, do Nascimento P (2011). Maintaining tissue perfusion in high-risk surgical patients: a systematic review of randomized clinical trials. Anesth Analg.

[42] Aya HD, Cecconi M, Hamilton M, Rhodes A (2013). Goal-directed therapy in cardiac surgery: a systematic review and meta-analysis. Br J Anaesth.

[43] Fenwick E, Wilson J, Sculpher M, Claxton K (2002). Pre-operative optimisation employing dopexamine or adrenaline for patients undergoing major elective surgery: a cost-effectiveness analysis. Intensive Care Med.

[44] Guest JF, Boyd O, Hart WM, Grounds RM, Bennett ED (1997). A cost analysis of a treatment policy of a deliberate perioperative increase in oxygen delivery in high risk surgical patients. Intensive Care Med.

[45] Bartha E, Davidson T, Hommel A, Thorngren KG, Carlsson P, Kalman S (2012). Cost-effectiveness analysis of goal-directed hemodynamic treatment of elderly hip fracture patients: before clinical research starts. Anesthesiology.

[46] Ebm CC, Sutton L, Rhodes A, Cecconi M (2014). Cost-effectiveness in goal-directed therapy: are the dollars spent worth the value?. J Cardiothorac Vasc Anesth.

[47] Bartha E, Davidson T, Brodtkorb TH, Carlsson P, Kalman S (2013). Value of information: interim analysis of a randomized, controlled trial of goal-directed hemodynamic treatment for aged patients. Trials.

[48] Jones AE, Troyer JL, Kline JA (2011). Cost-effectiveness of an emergency department-based early sepsis resuscitation protocol. Crit Care Med.

[49] Talmor D, Greenberg D, Howell MD, Lisbon A, Novack V, Shapiro N (2008). The costs and cost-effectiveness of an integrated sepsis treatment protocol. Crit Care Med.

[50] Pro CI, Yealy DM, Kellum JA, Huang DT, Barnato AE, Weissfeld LA, Pike F, Terndrup T, Wang HE, Hou PC (2014). A randomized trial of protocol-based care for early septic shock. N Engl J Med.

[51] McCleary L (2002). Using multiple imputation for analysis of incomplete data in clinical research. Nurs Res.

